# Endovascular management of colic artery pseudoaneurysm: A case report of successful intervention in a 59-year-old male with abdominal hematoma

**DOI:** 10.1016/j.radcr.2023.10.045

**Published:** 2023-11-17

**Authors:** Shivam Khatri, Rooshi Parikh, Matthew Smith, Joseph Friedman

**Affiliations:** a CUNY School of Medicine, New York, NY 10031, USA; b Department of Radiology, Jamaica Hospital Medical Center, Queens, NY 11418, USA

**Keywords:** Pseudoaneurysm, Inferior mesenteric artery, Colic artery, Embolization, Endovascular treatment

## Abstract

Visceral artery pseudoaneurysms, including inferior mesenteric artery pseudoaneurysms, are rare, occurring at an estimated incidence of 0.01%-0.2%. The literature reports only around 60 cases of inferior mesenteric pseudoaneurysm to date. The management of this condition lacks a consensus; nevertheless, coil embolization remains the preferred approach for stable patients. Here, we present a unique clinical scenario involving a 59-year-old male who underwent exploratory laparotomy for a retroperitoneal hematoma. Subsequently, he was diagnosed with an inferior mesenteric pseudoaneurysm, specifically affecting the left colic artery, and successfully managed using coil embolization.

## Introduction

Aneurysms are localized dilatation of a blood vessel in which all 3 layers of the blood vessel wall (intima, media, and adventitia) are dilated [1]. Aneurysms are most commonly seen within the aorta, although they can occur in any blood vessel [3]. Pseudoaneurysms, on the other hand, are false dilatation of blood vessels caused by damage to the arterial wall resulting in contained extravasation of blood [1]. Pseudoaneurysms are most commonly seen within peripheral arteries, which perfuse the extremities, such as the femoral artery [2]. They are less commonly seen within visceral arteries, which perfuse the abdominal organs, such as the splenic artery [2]. Aortic pseudoaneurysms are even less common [2].

Visceral artery pseudoaneurysms (VAPAs) is a broad term for any pseudoaneurysm that affects renal or splanchnic arteries [3]. VAPAs can be caused by disease pathology or iatrogenic injury from catheter-based interventions. They are relatively rare with an overall incidence rate of 0.01%-0.2% [3], [4], [5], [6]. Incidence of aneurysmal disease within visceral arteries is highest within the splenic artery at approximately 60% and lowest within the inferior mesenteric artery at approximately 1% [3,^5^,6].

VAPAs most often present with signs and symptoms of hemorrhage dependent on the ruptured vessel. Ruptured pseudoaneurysms of the renal artery may present with hematuria, while ruptured pseudoaneurysms of the celiac trunk, superior mesenteric artery, or inferior mesenteric artery may present with gastrointestinal bleeding, all in addition to hypotension and/or shock [7]. Treatment of VAPAs includes surgical, endovascular, percutaneous, and endoscopic options. The preferred approach is endovascular using some form of embolic material such as coils, liquid embolic, and/or gel foam [7]. We present a case of a 59-year-old male who initially underwent exploratory laparotomy during his first hospital visit for a retroperitoneal hematoma. However, he returned 2 days later with similar symptoms and was diagnosed with an inferior mesenteric artery pseudoaneurysm (IMAP). The interventional radiology team successfully treated the IMAP with coil embolization.

## Case presentation

A 59-year-old male with a past medical history of coronary artery disease, hypertension (controlled by metoprolol and lisinopril), hyperlipidemia, and previous sternotomy and valve replacement (prescribed warfarin with the last dose taken 2 days ago), presented to the emergency department (ED) with abdominal pain, dizziness, lightheadedness, nausea, and non-bloody vomiting. He denied smoking or alcohol use and had no prior colonoscopies or endoscopies. On admission, he presented with hypotension and tachycardia and was treated with IV fluids. A physical exam revealed a soft abdomen with left lower quadrant (LLQ) tenderness, rebound, and guarding, along with a non-incarcerated distended umbilical hernia. Lab results showed hyponatremia, hypocalcemia, and an elevated prothrombin time. A contrast-enhanced computed tomography (CT) scan of the abdomen and pelvis detected 2 adjacent soft tissue density masses in the left small bowel mesentery with an associated region of focal high density extending into the left pararenal space. The surgical team was consulted for an immediate diagnostic laparoscopy with possible exploratory laparotomy. After surgical exploration, the patient was diagnosed with a substantial hematoma involving the transverse and descending colon, although no active bleeding was observed. Throughout his hospitalization, there was a notable improvement in his hemoglobin and heart rate, however, his international normalized ratio (INR) remained elevated. Consequently, on the ninth day of hospitalization, he was discharged and prescribed a Lovenox bridge with warfarin. Instructions were given for him to follow up at the ambulatory care clinic in 1 week to monitor his INR levels.

**Fig. 1:**
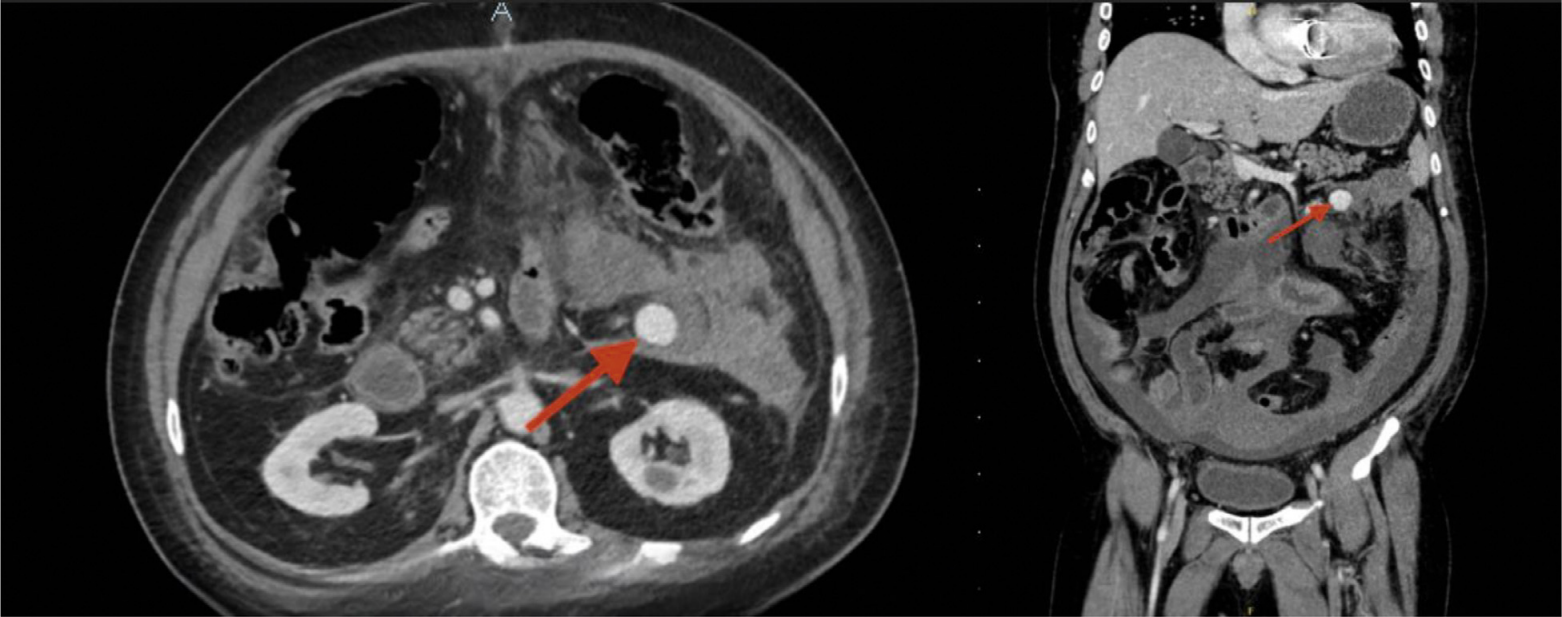
(A) Axial view representing pseudoaneurysm of the inferior mesenteric artery (red arrow). (B) Coronal view representing pseudoaneurysm of the inferior mesenteric artery (red arrow).

One day after discharge, the patient presented back to the ED with ongoing lower abdominal pain. During evaluation, he was found to have atrial fibrillation with rapid ventricular response, which was managed with rate-control measures. While in the ED, a CT scan of the abdomen and pelvis was performed, revealing resolving mesenteric hematomas. Additionally, a rounded hyperdensity in the left mid-abdomen raised suspicion for a pseudoaneurysm in a branch of one of the mesenteric arteries (Fig. 1).

The CT scan also indicated the interval development of free fluid within the abdomen and pelvis, potentially compatible with blood products. The following morning, interventional radiology (IR) was consulted to assess the feasibility of an angiogram, and based on its results, a decision would be made collaboratively between the surgical team and IR regarding the possibility of embolization or surgery.

**Fig. 2:**
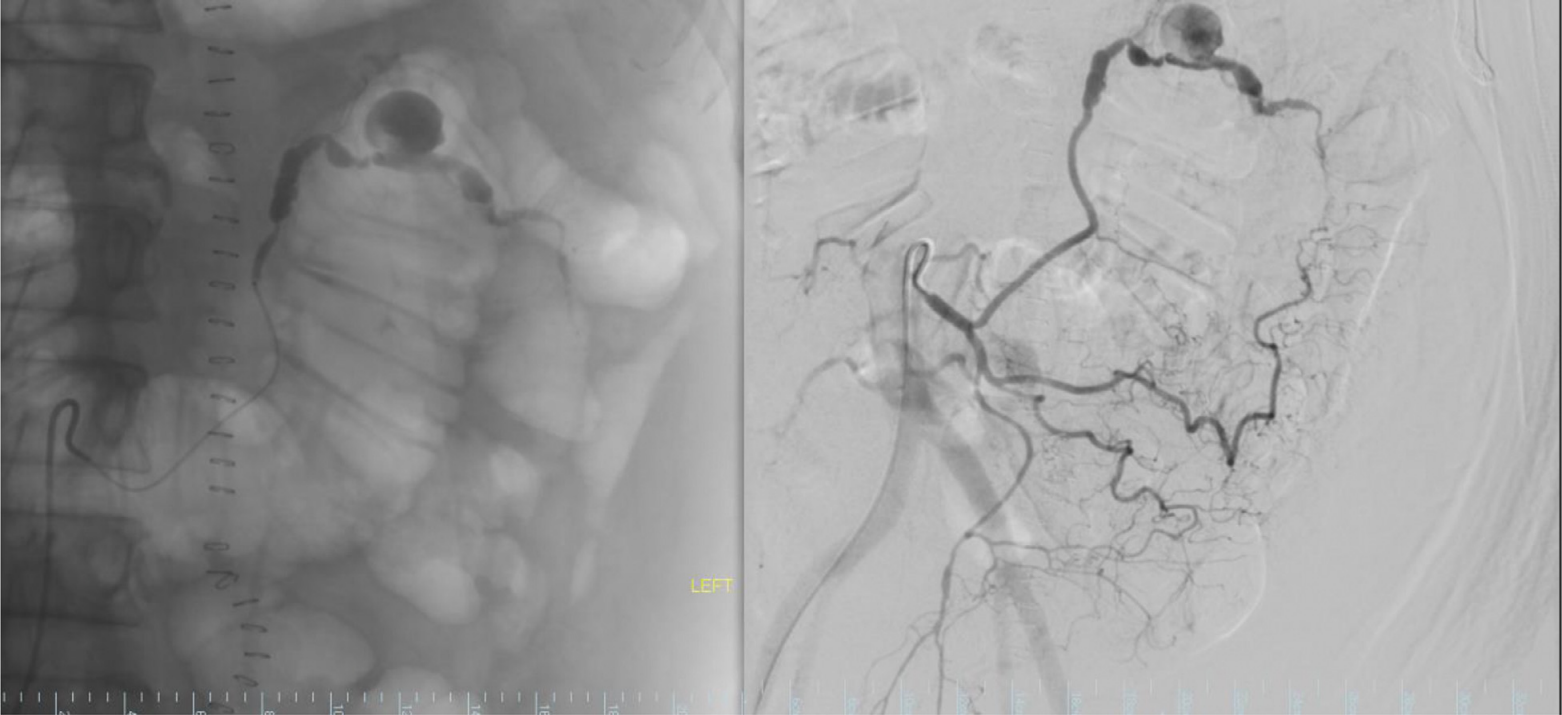
The left image is the fluoroscopy image of the distal segment of the ascending branch of the left colic artery exhibiting a pseudoaneurysm measuring 1.8 cm while the right image represents the digital subtraction angiography (DSA).

After prepping the skin with 2% chlorhexidine, the patient was positioned supine on the angiography table. Local anesthesia in the form of 1% lidocaine was administered. Using direct ultrasound guidance, a 21-gauge micropuncture needle was utilized to puncture the right common femoral artery, followed by the insertion of a 0.018 guidewire. Subsequently, the needle was exchanged for a 5 F micropuncture coaxial dilator. The inner dilator guidewire was removed, and a 0.035 j-tip guidewire was introduced. Next, the outer dilator was replaced with a 5F sheath. Employing the SOS catheter, we successfully catheterized the inferior mesenteric artery (IMA), allowing us to inject contrast and perform an IMA arteriogram. This revealed antegrade flow within the IMA and its branches. Notably, the distal segment of the ascending branch of the left colic artery exhibited abnormalities, characterized by areas of dilatation, stenosis, and a pseudoaneurysm measuring 1.8 cm (Fig. 2).

**Fig. 3:**
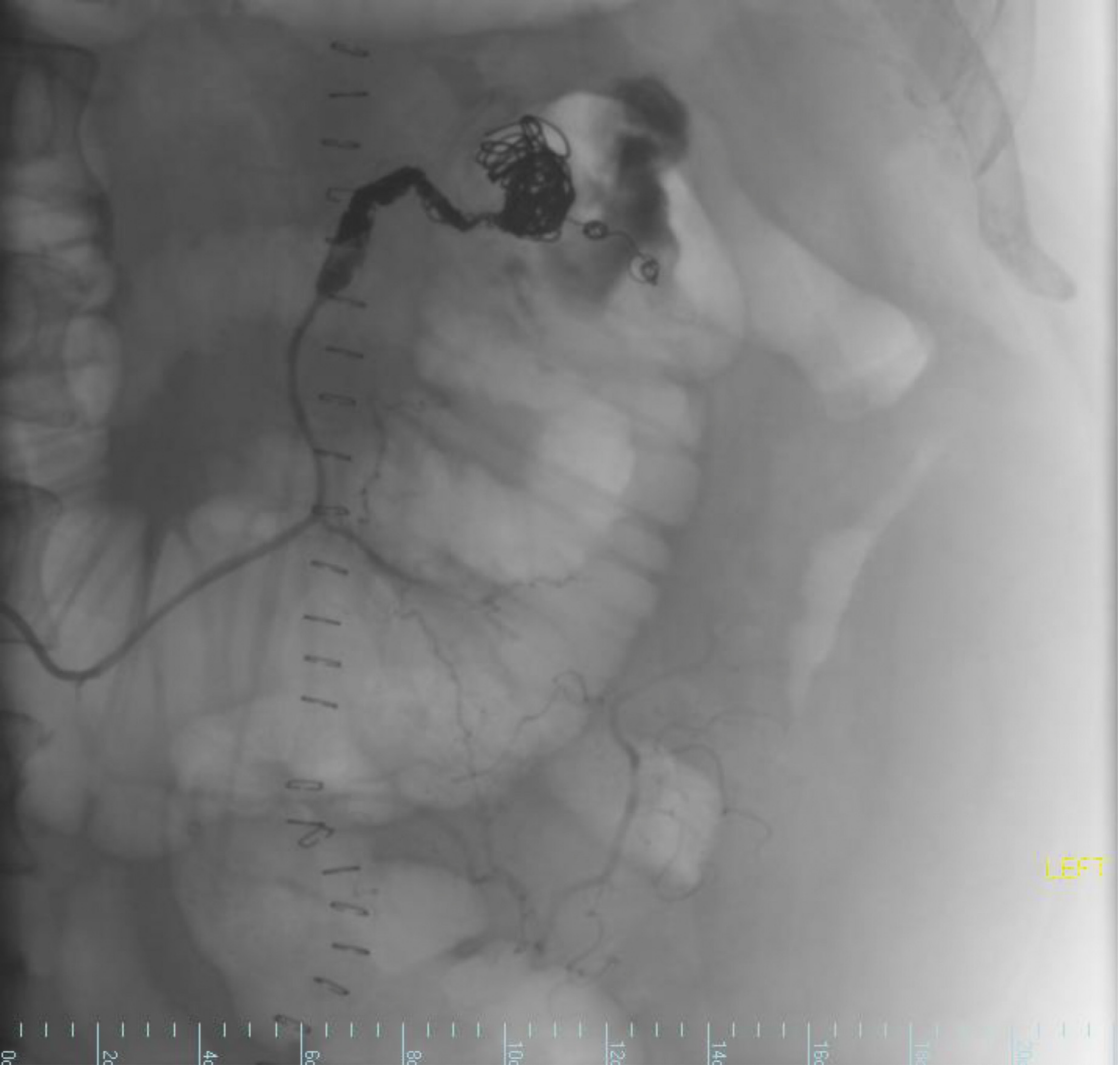
Fluoroscopy image showing coil embolization of IMA pseudoaneurysm and no further opacification of the abnormal segment.

After performing the angiogram, the case was reviewed with the surgical attending, who recommended coil embolization of the abnormal arterial segment. The patient was reminded about the risks associated with bowel ischemia and infarction, and the possibility of surgical exploration after embolization. Using a 2.8 F ProGreat microcatheter inserted through the SOS catheter, the ascending branch of the left colic artery was selected. Coil embolization was carried out distal to the pseudoaneurysm, using two 5 mm Cook Tornado microcoils. The pseudoaneurysm was then packed and coiled using a 12 mm x 60 cm Penumbra ruby coil standard, an 8 mm x 35 cm Penumbra ruby coil soft, and a 5 mm x 30 cm Penumbra ruby soft coil. A minimal amount of gel foam slurry was injected, followed by 45 cm and 30 cm Penumbra packing coils in the abnormal arterial segments proximal to the pseudoaneurysm (Fig. 3).

Subsequent arteriograms through the microcatheter and SOS catheter in the origin of the inferior mesenteric artery showed no further opacification of the abnormal segment or pseudoaneurysm. The SOS catheter was then repositioned to the celiac access, where an arteriogram revealed antegrade flow within the hepatic, left gastric, and splenic arteries, along with a replaced left hepatic artery. There was no opacification of the abnormal colic arterial segment or pseudoaneurysm. The SOS catheter was subsequently repositioned to the superior mesenteric artery, and an arteriogram showed antegrade flow within the superior mesenteric arteries and its branches, without opacification of the abnormal colic arterial segment or pseudoaneurysm. The catheter and sheath were removed, achieving hemostasis with the Mynx closure device and manual compression. The patient tolerated the procedure well and left the angiography suite in stable and satisfactory condition. The patient was discharged on hospital day 8 and was given outpatient follow-up with the surgical team.

## Discussion

IMAP is the rarest form of VAPAs with an incidence of roughly 1% [3,^5^,6]. A review done by Obara et al. found only about 54 cases of IMA aneurysms reported in the literature while another study reported around 60 cases, the majority of these cases being found in men between the age of 9-84 [8,9]. Most of these IMA aneurysms were located at the proximal trunk of IMA, and as a result, symptoms included asymptomatic pulsatile mass, dull abdominal pain, and shock when they ruptured [8]. The etiology of IMAPs can range from disease states, inflammation, infection, trauma, or iatrogenic injury [3]. Atherosclerosis, and similar causes of vessel wall degeneration, have been noted to be the most common causes of VAPAs [3,^8^,10]. Vasculitides such as Takayasu's disease, polyarteritis nodosa, and Behcet's disease have also been reported as possible causes [9,10]. The direct cause of IMAP in our patient is unknown but may be due, in part, to atherosclerosis from a history of hyperlipidemia or perhaps due to any trauma that occurred from the patient's previous exploratory laparotomy. Diagnosis of VAPAs can be made using abdominal ultrasound, however, contrast-enhanced CT angiography may provide the best visualization of morphology [8], [9], [10]. Between 20% and 50% of IMAP can present as ruptures [9].

Due to the rarity of IMAPs, the management and treatment approaches for this condition heavily rely on information gathered from other case reports. Marichal et al. presented a remarkably similar case to ours, wherein initial celiac and superior mesenteric arteriograms yielded negative results, leading to selective IMA arteriography. This imaging procedure demonstrated a left colic pseudoaneurysm similar to the source of the IMA pseudoaneurysm in our case, with rupture into the pancreatic duct and active duodenal hemorrhage. The authors opted for coil embolization as the treatment modality [11]. Similarly, Nagarajan et al. [8] reported a case involving a 45-year-old male who underwent exploratory laparotomy for hemoperitoneum, paralleling our case . Twenty days after the surgical exploration, the patient experienced bleeding per rectum, prompting the performance of contrast-enhanced CT and digital subtraction angiography, which revealed an IMA pseudoaneurysm communicating with the sigmoid colon [8]. As in our case, coil embolization was employed as the treatment strategy [8]. Borzelli et al. [12] reported yet another similar case of a VAPA, but one affecting the middle colic artery coming off the superior mesenteric artery. A giant VAPA was found incidentally on CT after the 70-year-old female patient presented to the ED complaining of right upper quadrant pain and a pulsatile periumbilical region [12]. After a multidisciplinary discussion, the medical team opted to perform a selective angiography and subsequent coil embolization, much in the same way as in our case [12].

A high risk of rupture and bleeding calls for a swift diagnosis of any pseudoaneurysm [13]. For this purpose, ultrasound offers an efficient and minimally invasive approach to imaging pseudoaneurysms. Duplex ultrasound is the preferred modality as it uses both a combination of B-mode and Doppler ultrasound [13]. B-mode ultrasound usually produces a hypoechoic or anechoic cystic-shaped structure situated next to the feeding artery [13]. This alone, however, is nondiagnostic. The additional use of Doppler imaging, which may produce a characteristic “ying-yang” sign due to systolic inflow and diastolic outflow, along with a typical “to-and-fro” waveform on spectral analysis, can help confirm the diagnosis [13].

Swift intervention, after quick diagnosis, is essential due to VAPA's high risk of rupture and a 25%-70% mortality rate [14]. Minimally invasive endovascular methods are favored, but optimal management lacks consensus, relying on case reports/series [8,9]. Catheters target and access the feeding vessel for embolic material deployment. Coils were used in our case, but gel foam slurry, glue, or thrombin are viable options [7,14]. Transcatheter embolization is the primary approach for stable patients, considering existing collaterals, but surgical intervention may become necessary [11]. Potential complications include femoral artery pseudoaneurysms, thrombosis, recurrent bleeding, or bowel ischemia/infarction [5].

Covered stent placement has been reported for ruptured VAPAs, especially when preserving the patency of the feeding vessel is necessary [11,15]. Notably, a large clinical study utilizing self-expanding stents for the elective treatment of visceral artery aneurysms demonstrated a 100% technical and clinical success rate [16]. However, certain criteria must be met for stent usage, such as ensuring sufficient vessel length on both sides of the pseudoaneurysm for an adequate seal [5]. While some studies recommend using self-expanding covered stents for treating tortuous arteries, balloon-expandable stents are preferred for straight arteries [5]. In select cases, a combined approach involving transcatheter embolization may be employed [5].

In cases of hemodynamic instability, open surgery may be preferred, involving arterial bypass, exclusion of the aneurysmal sac, or vessel ligature [5]. Operative intervention for IMA aneurysms requires a size greater than 2 cm proximally or 1 cm distally of the IMA [13]. Open surgery provides full visual confirmation, but with interventional radiology advancements, surgical options are less sought after.

## Conclusion

Inferior mesenteric pseudoaneurysms, particularly those affecting the colic artery, are exceedingly rare occurrences. While coil embolization stands as the preferred first-line treatment for most VAPAs, further insights from case reports and case series are essential for interventional radiologists to establish a consensus treatment for this uncommon condition.

## Ethical approval/clinical trial registration

Waived as per hospital policy.

## Patient consent

Written informed consent for publication was obtained from the patient prior to writing and submitting this case study.
